# Optical and Magneto-Optical Properties of Donor-Bound Excitons in Vacancy-Engineered Colloidal Nanocrystals

**DOI:** 10.1021/acs.nanolett.1c01818

**Published:** 2021-07-14

**Authors:** Francesco Carulli, Valerio Pinchetti, Matteo L. Zaffalon, Andrea Camellini, Silvia Rotta Loria, Fabrizio Moro, Marco Fanciulli, Margherita Zavelani-Rossi, Francesco Meinardi, Scott A. Crooker, Sergio Brovelli

**Affiliations:** † Dipartimento di Scienza dei Materiali, Università degli Studi di Milano-Bicocca, via Cozzi 55, IT-20125 Milano, Italy; ‡ Dipartimento di Energia, Politecnico di Milano, IT-20133 Milano, Italy; § National High Magnetic Field Laboratory, Los Alamos National Laboratory, Los Alamos, New Mexico 87545, United States

**Keywords:** nanocrystal quantum dots, electronic doping, sulfur vacancy, bound exciton, magneto-optics, spectro-electrochemistry

## Abstract

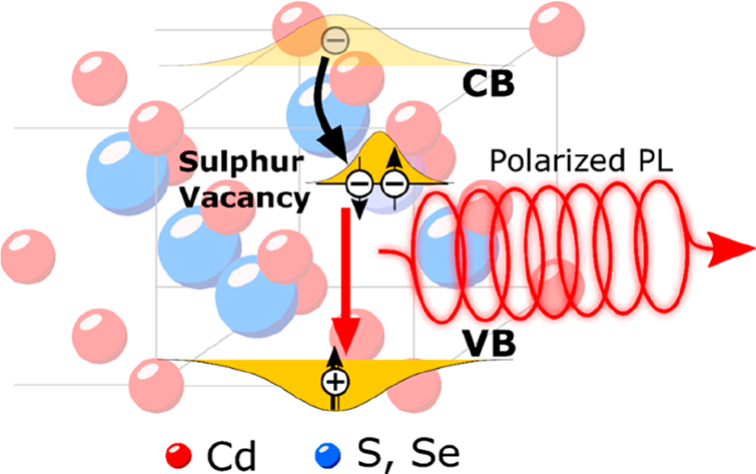

Controlled insertion of electronic states within the band gap of semiconductor nanocrystals (NCs) is a powerful tool for tuning their physical properties. One compelling example is II–VI NCs incorporating heterovalent coinage metals in which hole capture produces acceptor-bound excitons. To date, the opposite donor-bound exciton scheme has not been realized because of the unavailability of suitable donor dopants. Here, we produce a model system for donor-bound excitons in CdSeS NCs engineered with sulfur vacancies (*V*_S_) that introduce a donor state below the conduction band (CB), resulting in long-lived intragap luminescence. *V*_S_-localized electrons are almost unaffected by trapping, and suppression of thermal quenching boosts the emission efficiency to 85%. Magneto-optical measurements indicate that the *V*_S_ are not magnetically coupled to the NC bands and that the polarization properties are determined by the spin of the valence-band photohole, whose spin flip is massively slowed down due to suppressed exchange interaction with the donor-localized electron.

Colloidal semiconductor nanocrystals (NCs) are intensively investigated functional materials because of their tunable physical properties and solution-phase processability that make them promising candidates for several optoelectronic and photonic devices.^1−6^ Doping NCs with impurities having a different valence with respect to the host atom that they replace (i.e., heterovalent doping)^7−15^ offers the possibility to engineer the number and type of carriers in a NC and has become an established paradigm for achieving technologically relevant functionalities, including a large Stokes-shift between the emission and the absorption spectra,^7−9^ extended luminescence lifetimes,^7−10^ photomagnetic behaviors,^8,16,17^ and enhanced electrical transport.^11,18−20^ Compelling examples of heterovalent-doped NCs have been produced using *p*-type impurities in II–VI semiconductors, such as *d*^10^ coinage metals (Cu^+^, Ag^+^, or Au^+^),^7−10,21^ which introduce localized acceptor states pinned 300–700 meV above the NC valence band (VB). Upon photoexcitation of the NC host, such states rapidly capture the photohole leading to the formation of an “acceptor-bound” exciton with the CB electron, whose radiative recombination gives rise to size-tunable intragap luminescence.^7^ Furthermore, transient oxidation of such *d*^10^ impurities to their paramagnetic *d*^9^ configuration following hole capture confers photomagnetic character to the NC.^8,17^ Despite such an in-depth knowledge of hole management in doped NCs, very little is known about the possibility of manipulating the electron dynamics through the incorporation of donor impurities that introduce deep localized states below the CB of the host NC. Although conceptually analogous to hole-management schemes, the realization of donor-bound excitons using aliovalent elements adopted to *n*-doped NCs (e.g., Al^3+^ and In^3+^ in II–VI or IV–VI compounds) is challenging due to the natural propensity of such cations to produce shallow donor states that inject electrons directly in the CB,^19,22−28^ leading to enhanced electron transport without modifying the decay pathway for band edge (BE) excitons. This limits our flexibility in engineering carrier dynamics in doped systems which might provide the key for new physical behaviors toward new multilevel electronic or photonic schemes as well as advanced spintronic devices.

Here, we aim to contribute to this goal by realizing and investigating the optical and magnetic properties of II–VI NCs featuring donor-bound excitons. With this aim, we realized a model system adopting a vacancy engineering strategy that exploits the propensity of metal sulfides to present sulfur vacancies (*V*_S_) that introduce a localized level pinned about 1 eV below the CB.^29−31^ More specifically, CdS bulk crystals and colloidal particles have been shown to emit self-activated intragap luminescence emerging from a Lambe-Klick mechanism^32^ where sulfur vacancies act as donors^33^ following the reaction *V*_S_ ⇌ *V*_S_^*+*^ + *e*_CB_^–^ with the Fermi energy for the first ionization being located ∼700 meV below the CB (the doubly charged *V*_S_^2+^ vacancy is a very deep trap for electrons and spontaneously converts to *V*_S_^+^ upon attracting an electron from the VB). Following photoexcitation, ultrafast capture of *e*_CB_^–^ by *V*_S_^+^ temporarily reduces them to *V*_S_^0^ centers that can either decay nonradiatively or recombine with the VB photohole producing the characteristic intragap luminescence.^29−31^ This makes NCs with sulfur vacancies interesting model systems to explore the implications of donor states in the optical and magneto-optical properties of NCs, possibly suggesting strategies to introduce new functionalities by design. In this framework, vacancy engineering is a powerful strategy to unlock thermodynamically unfavored chemical processes,^34^ to manipulate the physical properties of nanostructures,^18,19,35−38^ and to switch from the excitonic to the plasmonic regime in heavily vacancy-doped NCs.^39,40^ Here, to preserve the excitonic behavior, we synthesized a set of alloyed CdSeS NCs with controlled concentrations of *V*_S_ (hereafter indicated as *V*_S_/CdSeS NCs), whose physical properties we studied via continuous-wave and time-resolved photoluminescence (PL) measurements as a function of temperature, side-by-side with transient transmission (TT), spectro-electrochemistry (SEC), magnetic circular dichroism (MCD), and circular-polarization resolved magneto-PL. Our results demonstrate the ultrafast (∼1.2 ps) formation of a donor-bound exciton responsible for a long-lived emission (indicated as *V*_S_-PL) with size-tunable energy, consistent with the *V*_S_-state being pinned to the host CB. SEC and temperature-controlled PL experiments reveal that electrons in *V*_S_ are nearly unaffected by nonradiative trapping and that coupling to phonons plays a major role in the thermal quenching of the *V*_S_-PL, whose suppression at low temperatures boosts the emission efficiency to ∼85%. Circularly polarized magneto-PL reveals that the *V*_S_-PL polarization is determined by the spin of the VB hole, and because of the strongly reduced electron–hole overlap with the *V*_S_-localized electron the exchange interaction contribution to the hole spin flip is strongly suppressed, resulting in hole spin-flip times over 1 order of magnitude longer than in undoped NCs.

## Results and Discussion

**Figure 1:**
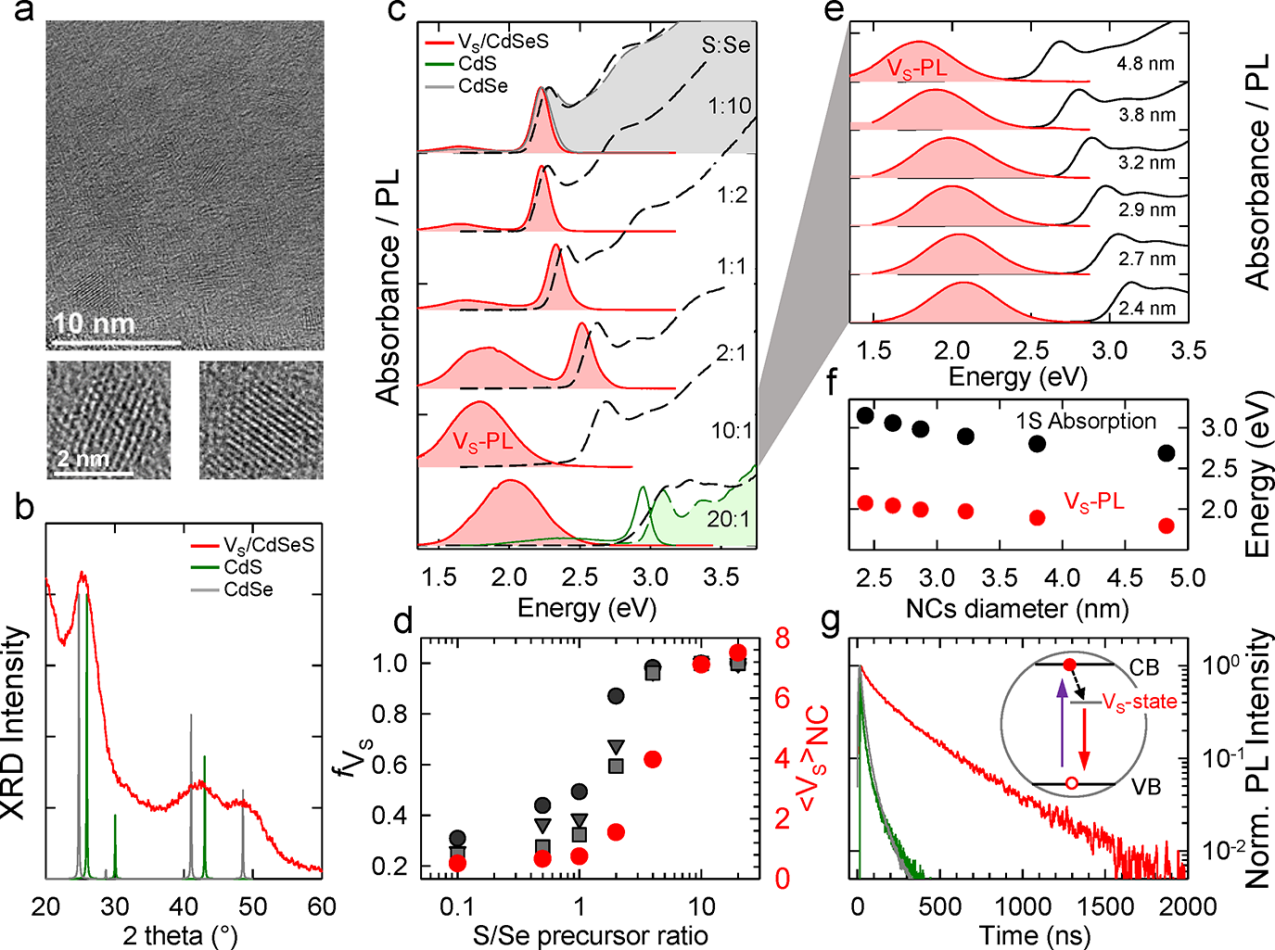
(a) TEM images of representative *V*_S_/CdSeS NCs featuring mean radius of 2.9 ± 0.7 nm. (b) XRD pattern of the same NCs compared to pristine zincblende CdS (green line) and CdSe (gray line). (c) Optical absorption (black dashed lines) and PL spectra (red curves) of *V*_S_/CdSeS NCs synthesized using increasing amounts of DDT as sulfur precursor (from top to bottom). Pure CdSe (gray line) and CdS (green line) NCs absorbance and PL profiles are reported as references at the top and the bottom of the plot, respectively. All NC samples have comparable diameter of ∼4.4 nm. (d) Fraction of particles in the NCs ensemble collected at different stage of growth synthesis (gray markers for intermediate aliquots, red markers for final) presenting sulfur vacancies, extracted from the relative weights of the residual BE-PL and *V*_S_-PL of panel (c). The data are reported as a function of the molar ratio of selenium and sulfur precursors used in the NC synthesis together with the average number of sulfur vacancies per NC. (e) Dependence of the optical properties (absorbance and PL) of *V*_S_/CdSeS NCs on the size of the NCs. NCs were prepared with S/Se ratio = 10. (f) Energy of the 1S absorption peak and *V*_S_-PL band as a function of the particle diameter. (g) PL decay traces of the *V*_S_-PL (red curve) compared with pristine CdS (green curve) and pristine CdSe (gray curve). The inset depicts the recombination process.

The preparation of *V*_S_/CdSeS NCs and control CdSe and CdS NCs is described in the Methods section of the Supporting Information. Transmission electron microscopy (TEM) images of representative *V*_S_/CdSeS NCs are reported in Figure 1a, showing spherical particles with average size of 2.9 ± 0.7 nm (see Figure S1 for the size distribution statistic). The XRD pattern (Figure 1b) shows zincblende crystal structure.^41,42^ The width of the peaks and their intermediate position between the diffraction maxima of zincblende CdSe and CdS suggests an alloyed structure in agreement with the optical absorption data, showing a progressive blue shift of the 1S absorption energy from that of pure CdSe NCs to that of CdS NCs of comparable size (2.5 nm, Figure 1c). The optical spectra of NCs produced with different S/Se ratio collected at increasing reaction time (Figure S2) evidence that alloying occurs already at the nucleation stage and that the initial clusters have composition dictated by the S/Se precursor ratio. Consistent with previous results,^43,44^ the electron paramagnetic resonance (EPR) spectrum of *V*_S_/CdSeS NCs reported in Figure S3 shows the characteristic resonance signal at *g* = 2.001 ± 0.001 due to sulfur vacancies.

More importantly, alloying modifies the luminescence properties of the NCs, with the PL evolving from the BE emission of CdSe or CdS NCs (Figure 1c) to the broad intragap *V*_S_-PL of *V*_S_/CdSeS NCs, Stokes-shifted from the absorption onset by ∼1 eV. The residual BE-PL in *V*_S_/CdSeS NCs produced with intermediate S/Se ratios is ascribed to a subpopulation of NCs in the ensemble without sulfur vacancies. This agrees with TT measurements (*vide infra*) that reveal an electron capture time in the *V*_S_-state of ∼1.2 ps, which instantaneously quenches the BE-PL. The average fraction of doped particles *f*_*V*_S__ versus the S/Se precursor ratio extracted following the procedure reported in the Supporting Information is shown in Figure 1d, which further indicates that in “fully doped” NC ensembles (*f*_*V*_S__ > 0.99), each particle contains an average of seven sulfur vacancies. The analysis of the absorption spectral position using Végard’s Law^45,46^ suggests that for S/Se = 10, NCs have the approximate composition CdSe_0.2_S_0.8_. The estimation of the number of *V*_S_ using ICP-AES compositional analysis is challenging due to the release of H_2_S that leads to systematic underestimation of the sulfur content. Most importantly, for *V*_S_-NCs produced using S/Se = 20 (with composition CdSe_0.1_S_0.9_, Figure 1c), the *V*_S_-PL is dramatically different from the emission of pure CdS NCs, thus confirming that alloying is necessary to engineer the formation of sulfur vacancies. A further look at Figure S2 reveals that the first aliquots produced at any S/Se precursor ratio exhibit predominant *V*_S_-PL and that the BE emission becomes dominant at later growth stages, suggesting that the number of *V*_S_ per NC drops as the surface/volume ratio decreases. To investigate the location of *V*_S_ in the inner part or on the surfaces of our NCs, we thus employed the colloidal atomic layer deposition (c-ALD) method to add a single sulfur monolayer to fully doped NCs exhibiting exclusively the vacancy related PL (see Methods in Supporting Information and Figure S4). The c-ALD treatment results in the emergence of the BE-PL accompanied by the decrease of the vacancy related emission, suggesting that surface sulfur vacancies are saturated through postsynthesis sulfuration and that some *V*_S_ might also be located in the inner part of the NCs. Because of its mixed vacancy-host character, the *V*_S_-PL can be tuned spectrally by shifting the CB of the NCs as highlighted in Figure 1e where we report the PL spectra of NCs with increasing size produced using S/Se = 10. The energies of the 1S absorption peak and *V*_S_-PL follow a nearly identical trend as a function of the particle radius diameter (Figure 1f). Considering the substantial difference between the electron and hole effective masses, this indicates that the *V*_S_ state is pinned to the CB. Consistent with the recombination mechanism emerging from the spectral behavior, time-resolved PL measurements reveal lengthening of the decay dynamics of *V*_S_/CdSeS NCs with respect to control NCs (Figure 1g). Specifically, the PL decay of CdSe and CdS NCs are multiexponential with effective exciton lifetime of ∼20 ns (extracted as the time needed to reduce the initial signal by a factor *e*) as commonly observed for unshelled NCs, whereas *V*_S_/CdSeS NCs exhibit 8-fold longer decay time (<τ> ≈ 180 ns) due to the substantially smaller spatial overlap between the VB photohole and the electron localized in the *V*_S_ state.

**Figure 2:**
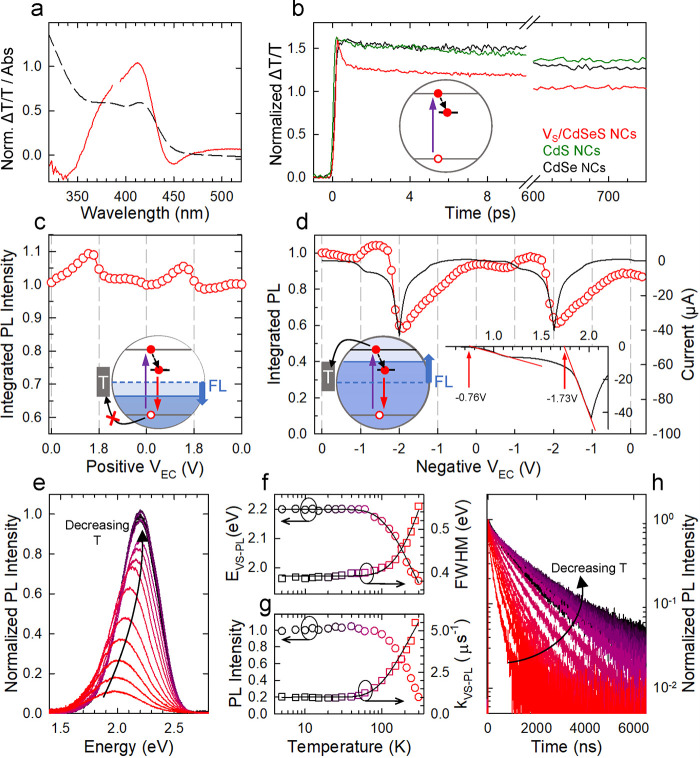
(a) Linear absorption (dashed black curve) and transient transmission (TT) spectrum collected at 300 fs pump–probe delay time (red line, *E*_EXC_ = 3.05 eV, power density of 100 nJ cm^–2^) of *V*_S_/CdSeS NCs. (b) Comparison between the bleaching dynamics of the 1S absorption peak of the *V*_S_/CdSeS NCs reported in (a) and conventional CdSe and CdS NCs (gray and green line, respectively). All dynamics are normalized to their initial values. In all cases, the bleaching dynamic occurs at pumping fluences well below the multiexciton regime under intense stirring, which excludes artifacts due to photocharging or multiexcitonic processes such as nonradiative Auger recombination. Integrated PL intensity of *V*_S_/CdSeS NCs during two successive (c) positive (*V*_EC_ from 0 to +1.8 V) and (d) negative (*V*_EC_ from 0 to −2 V) electrochemical scans (potential step 0.1 V). The black line in (d) is the corresponding current flowing through the NC film that is further magnified in the inset (first scan) to better highlight the current onset potentials. Schematic depiction of the effect of lowering (raising) the Fermi level (FL) under positive (negative) *V*_EC_ values are also reported as insets, showing suppressed hole (electron) trapping and direct electron injection in the *V*_S_-state and CB under increasing negative *V*_EC_. (e) PL spectra of *V*_S_/CdSeS NCs (S/Se = 10:1) at decreasing temperature from 300 to 5 K as indicated by the black arrow. (f) Spectral position (hollow circles) and full width at half-maximum (hollow squares; fwhm) of the PL peak together with the respective fitting curves to eq 1 and eq 2. (g) Integrated PL intensity (hollow circles) normalized to its value at 5 K together with the PL decay rate (hollow squares) extracted from the decay curves of the same NCs as a function of temperature shown in (h). The black curve is the fit of the PL decay rate vs *T* according to eq 3. All measurements are excited at 3.05 eV.

Independent confirmation of the proposed mechanism is provided by TT measurements that further enable us to evaluate the electron capture time by the *V*_S_ state. The TT spectra of *V*_S_/CdSeS NCs (Figure 2a) show a strong positive bleaching band of the 1S absorption peak due to state filling of the doubly degenerate CB.^47^ Notably, substantial difference between pure and *V*_S_/CdSeS NCs appears in the bleaching dynamics, which is direct consequence of the depletion channel for the CB associated with electron localization (Figure 2b). Specifically, CdSe and CdS NCs follow the typical slow bleaching dynamics,^48,49^ whereas *V*_S_/CdSeS NCs exhibit an ultrafast bleaching recovery (∼1.2 ps) due to rapid electron capture in the *V*_S_-state. This suggests that other CB depletion mechanisms such as electron trapping might play a minor role in the decay mechanism. Nonetheless, the emission quantum yield at room temperature (Φ_*V*s-PL_ = 17 ± 2%) indicates that other nonradiative decay pathways are competing with the radiative recombination of the bound excitons.

To investigate the nature of such processes, we performed spectro-electrochemistry (SEC) experiments that enable one to monitor the emission of NCs while their Fermi Level (FL) is tuned by an electrochemical potential (*V*_EC_) and thereby to probe the effect of surface traps on the PL intensity.^50−54^ Specifically, the application of a positive *V*_EC_ (corresponding to lowering the FL) depletes the NCs of excess electrons in undercoordinated surface sites or dangling bonds that might act as nonradiative traps for photoholes and, concomitantly, it activates surface electron traps, thus possibly enhancing nonradiative losses.^55,56^ Contrarily, the application of a negative *V*_EC_ (corresponding to raising the FL) provides electrons for electron-poor sites, thus suppressing electron trapping and creating electron-rich centers that might capture the photoholes. Therefore, brightening versus bleaching effects of the PL intensity under positive versus negative *V*_EC_ provide an indication on the dominant nonradiative trapping channel at equilibrium (*V*_EC_ = 0 V).^57^ In our case, the application of positive *V*_EC_ leads to a weak, ∼10%, PL brightening (Figure 2c), indicating passivation of hole traps and that electron traps do not quench donor-bound excitons, which suggests that after localization in the *V*_S_ state, electrons are less affected by trapping losses, similarly to localized photoholes in copper-doped NCs.^55^

In contrast, applying negative *V*_EC_ has substantial impact on the emission intensity of *V*_S_/CdSeS NCs, as shown in Figure 2d for two successive scans from *V*_EC_ = 0 to −2 V. The strongest effect is observed for *V*_EC_ < −1.8 V, where the PL intensity undergoes ∼55% drop with respect to the equilibrium conditions. This is ascribed to direct injection of electrons in the CB causing state filling of the 1S state and possibly activating nonradiative Auger decay via the negative trion pathway.^56,58^ Consistently, such a PL drop is accompanied by the increase of the transmitted excitation beam intensity at 3.05 eV (corresponding to the 1S absorption peak, Figure S5) and by the increase of the current flowing through the NC film. The analysis of the current versus voltage curves (inset of Figure 2d) reveals that the onset for electron injection in the CB occurs at *V*_EC_ = −1.73 V, corresponding to nearly half the bandgap energy of the *V*_S_/CdSeS NCs (*E*_g_ = 3.2 eV), which suggests that the FL in equilibrium conditions is positioned near the center of the forbidden gap, consistent with the low doping level inferred from the analysis of the optical spectra. Interestingly, we notice a weak (∼5%) reproducible intensification of the *V*_S_-PL for *V*_EC_ ∼ −1 V, which might originate from the passivation of electron traps active in unperturbed NCs and from electrical injection of electrons directly in the intragap *V*_S_-state. This scenario finds support in the measurable increase of the current with onset at *V*_EC_ ∼ −0.76 eV, nearly 1 eV below the onset of electron injection in the CB, which matches the ∼1 eV Stokes shift.

Next, we moved to investigating the effect of phonon coupling on the recombination of donor-bound excitons in *V*_S_/CdSeS NCs using temperature-controlled PL measurements. As shown in Figure 2e,f, upon lowering the temperature, the PL intensifies, narrows, and progressively shifts toward higher energy, consistent with the gradual widening of the energy gap of the semiconductor host. In analogy with previously reported doped NCs,^55,59^ the width of the *V*_S_-PL at low temperature is dictated by the coordination inhomogeneities of the *V*_S_ sites, which therefore experience slightly different crystal fields and lead to the observed broad PL peak also at cryogenic temperatures. Concomitantly, the integrated PL intensity (Figure 2g) shows a 5-fold increase, reaching saturation at *T* ∼ 100 K due to the gradual suppression of nonradiative thermal quenching. On the basis of the room-temperature value on the same sample, we estimate a low-temperature emission efficiency of Φ_PL_ ∼ 85%. As shown in Figure 2h, the emission kinetics show comparable lengthening with the decay rate dropping from *k*^300 K^ = 5.5 μs^–1^ (τ ∼ 180 ns) to *k*^T^ = 1 μs^–1^ (τ ∼ 1.0 μs) for *T* ≤ 60 K, which corresponds to the radiative decay rate (*k*_rad_) of donor-bound excitons in *V*_S_/CdSeS NCs. This effect occurs without changes of the zero-delay PL intensity, suggesting that the NCs are unaffected by thermally activated ultrafast trapping mechanisms and that Φ_PL_^300 K^ is mainly limited by electron–phonon coupling. To identify the phonon mode responsible for the thermal quenching, we analyzed the temperature dependence of the PL energy (*E*_*V*_S__ – PL) and FWHM using the equations^60^
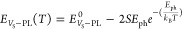
1
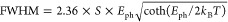
2where *k*_B_ is the Boltzmann constant, *S* is the Huang–Rhys factor, and *E*_ph_ is the energy of the phonon mode. The global fit of the experimental data yields *S* = 5.1 and *E*_ph_ = 29 meV, which is close to the energy of the longitudinal optical phonon modes of CdS and CdSe (35 and 25 meV, respectively). Independent confirmation of this assignment comes from the activation energy for nonradiative decay, *E*_A_ = 27 meV, extracted from the analysis of the PL lifetimes versus *T* (Figure 2g) though the equation, *k* = *k*_RAD_ + *k*_NRAD_(*T*) that neglects ultrafast surface trapping and expresses the nonradiative decay rate *k*_NRAD_(*T*) by the standard displaced harmonic oscillator model^60^. Interestingly, analogously to what observed for NCs featuring acceptor-bound excitons (e.g., CdSe doped with coinage metals),^55,59^ the analysis of the PL kinetics versus temperature of *V*_S_/CdSeS NCs shows no signature of the characteristic ultralong lived emission due to the recombination of so-called dark-excitons^61,62^ commonly found in undoped CdSe NCs at cryogenic temperatures, thus suggesting that also donor-bound exciton does not possess a low energy dark exciton state.

**Figure 3:**
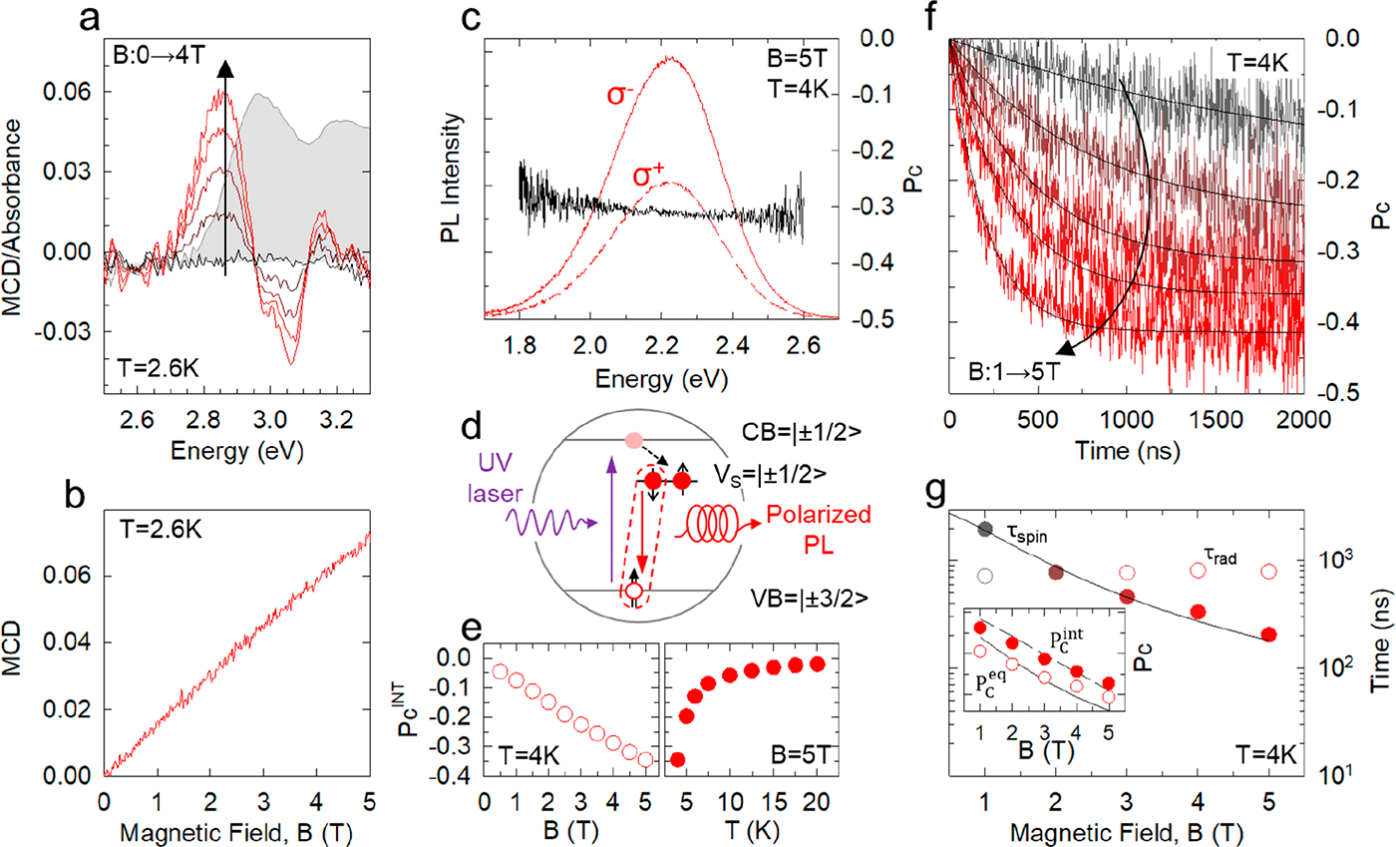
(a) Magnetic circular dichroism (MCD) spectra of *V*_S_/CdSeS NCs at *T* = 2.6 K upon increasing the external magnetic field, *B*, from 0 to 4 T (black to red lines). The linear absorption spectrum is reported as a gray shading. (b) MCD signal at 2.85 eV as a function of *B*. (c) Left- (σ^–^) and right-circularly polarized (σ^+^) PL spectra (red lines) of *V*_S_/CdSeS NCs at *T* = 4 K and *B* = 5 T and corresponding circular polarization degree (black line). (d) Scheme of the circularly polarized emission process dominated by the spin projection of the VB hole. (e) Magnetic- (left plot) and temperature-dependence of *P*_C_^INT^ (right plot). (f) Time-resolved *P*_C_ at increasing magnetic field from 1 to 5 T. Black lines are the results of the fitting procedure using eq 3. (g) Magnetic field dependence of τ_spin_ and of the radiative PL decay time, τ_rad_ (filled and empty markers, respectively). Black lines are the results of the fitting procedure with eq 4. Inset: Magnetic field dependence of *P*_C_ obtained from the analysis of the PL spectra (*P*_C_^int^, filled circles) and of the data reported in panel (f) (*P*_C_^eq^, empty circles), together with their fitting curves to (eq S3 and eq S4 in the Supporting Information).

Finally, we proceed with analyzing the magneto-optical properties of *V*_S_/CdSeS NCs. In Figure 3a, we report the MCD spectra of *V*_S_/CdSeS NCs as a function of the applied magnetic field *B*, showing the progressive increase of the typical derivative-like MCD signal associated with the Zeeman splitting of the 1S absorption peak. The almost perfect linear growth of the MCD upon increasing *B* (Figure 3b), together with its temperature-independence (not shown), indicate that the *V*_S_^+^ states are not magnetically coupled to the BE states.

Figure 3c reports the right and left circularly polarized PL spectra at *T* = 4 K and *B* = 5 T (σ^+^ and σ^–^, respectively) of the same *V*_S_/CdSeS NCs. The magnetic field clearly polarizes the PL, enhancing its σ^–^ component, in agreement with previous reports on CdSe,^63^ CdSe/CdS^64−66^ and CdSe/ZnS NCs.^67^ To quantify the polarization, we define the circular polarization degree, , where *I*^±^ is the intensity of the σ^±^ component of the PL. As shown in Figure 3c, *P*_C_ is almost constant over the PL emission band confirming that the PL arises from a single recombination process and allowing us to consider the spectrally integrated polarization degree, . The origin of the circularly polarized magneto-PL can be understood considering the scheme in Figure 3d. When not excited, the NC features a singly occupied *V*_S_^+^ level with angular momentum projection *M* = ±1/2. Following photoexcitation, photoelectron capture leads to its reduction to *V*_S_^0^, which is doubly occupied by counter-aligned electrons, whereas the corresponding photohole (*M* = ±3/2) resides in the VB. Since two localized electrons with opposite spins are available to recombine but selection rules only allow recombination between electrons and holes featuring counter aligned spins (Δ*M*= ± 1), the observed circularly polarized PL is direct consequence of the hole spin orientation.^7,66^ The application of an increasingly stronger *B* widens the Zeeman splitting between the hole spin sublevels (Δ*E*_Z_ = −2|*M*|*g*_h_μ_B_B, where *g* is the Landé-factor of the hole and μ_B_ is the Bohr magneton) unbalancing their relative thermal population at low temperatures, as evidenced by the growth of *P*_C_^int^ with increasing *B* (at *T* = 4 K) and also with decreasing temperature (at *B* = 5 T, Figure 3e). To investigate the dynamics of such a process, we measured the time-resolved *P*_C_ (Figure 3f) as a function of *B*. The growth of *P*_C_ over time is evidence of the hole thermalization to the lowest energy spin sublevel. At all fields, the experimental curves are well reproduced using the equation
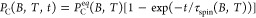
3where *P*_*C*_^eq^ is the degree of circular polarization when the thermal equilibrium between the spin sublevels is reached and τ_spin_(*B*,T) is the temperature- and magnetic field-dependent hole spin-flip time. It is worth noting that the *B*-dependence of τ_spin_ is well reproduced by the quadratic expression
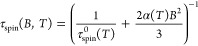
4where 1/τ_spin_^0^(T) is the hypothetical spin-flip time at *B* = 0 T and α(T) is the coefficient that describes the spin-flip mechanism as a two-phonon-mediated process, involving an intermediate ±1/2 virtual state (Figure 3g).^64,66,68^ When compared to the respective radiative decay time τ_rad_ (Figure 3g and Figure S6), τ_spin_ quickly shortens with increasing *B*, becoming faster than τ_rad_ for *B* > 2 T. This indicates that in these conditions the majority of the PL decay occurs when the maximum equilibrium polarization *P*_C_^eq^ is reached. This aspect is quantified by the dynamical factor, DF, defined as τ_rad_/(τ_rad_ + τ_spin_) (Figure S7) which undergoes a 3-fold increase and approaches unity with increasing *B*. On the basis of the obtained DF and using the model reported in ref (66), we adequately fitted the *B*-dependent trend of both *P*_C_^eq^ and *P*_C_^int^ (see also Supporting Information) obtaining a hole Landé *g*-factor of −0.91, consistent with literature reports.^7,66,69−73^

Importantly, we notice that for any applied *B*, τ_spin_ of *V*_S_/CdSeS NCs is ∼10-fold slower than reported values for analogous chalcogenide NCs in which the VB photohole is coupled to two electrons in the delocalized CB (and hence the PL helicity is determined by the hole spin), such as spherical CdSe (τ_spin_ = 0–10 ns^74^), core/shell CdSe/CdS NCs (τ_spin_ = 0–60 ns^64,66^), nanorods (τ_spin_ = 0–10 ns^75^), and nanoplatelets (τ_spin_ = 0–30 ns^76,77^) that feature stable negative trions at cryogenic temperatures. Considering that the number of carriers in photoexcited *V*_S_/CdSeS NCs is also three (two electrons in the *V*_S_^0^ state and one VB hole), such a remarkably slower hole spin flip time is ascribed to the substantially lower spatial overlap of the electron and hole wave functions, leading to strongly reduced spin flip by electron–hole exchange via the Bir–Aronov–Pikus mechanism.^78−80^ Specifically, in the case of *V*_S_/CdSeS NCs the strong electron localization in the deep *V*_S_ state minimizes the spatial overlap with the hole wave function that is fully delocalized in the particle volume, consistent with the μs-long lifetime of the donor-bound exciton (at low *T*, see Figure 2h). On the other hand, the electron–hole overlap in CdSe/CdS NCs is only partially reduced with respect to core-only systems due to the quasi-type II band-alignment that causes the spread of the electron wave function in the shell region (i.e., e-h overlap ∼0.1–0.5 with particle radius of 1.5 nm and shell thickness of 8–10 nm).^61,64,66^ Although detailed study of this effect goes beyond the aim of this work, the observed lengthening of the hole spin-flip time in *V*_S_/CdSeS NCs might suggest a possible route to engineer the spin dynamics of colloidal nanocrystals via vacancy engineering.

In conclusion, we have demonstrated a vacancy engineering scheme to realize a model system for NCs doped with donor impurities. The resulting donor-bound exciton regime was investigated by complementary optical and magneto-optical spectroscopies, highlighting a distinctive nearly trapping-free decay mechanism and intriguing magneto-optical properties determined by the spin dynamics of the delocalized photohole. Overall, these results suggest a possible strategy to widen the applicability of doped NCs for realizing new optical and magnetic schemes as well as for creating functional building blocks for advanced nanocrystal-based metamaterials.

## Supporting Information Available

Detailed description of the NC synthesis and experimental methods; characterization data such as NCs size distribution, optical absorption and PL spectra, EPR spectra, evaluation of the average number of vacancies per NCs, and theoretical calculation of circular polarization degree (PDF)
