# Supplementary material for: Optical and Magneto-Optical Properties of Donor-Bound Excitons in Vacancy-Engineered Colloidal Nanocrystals
Source: Nano Lett. 2021 Jul 14;21(14):6211–9. doi: 10.1021/acs.nanolett.1c01818 (PMC8397387; doi:10.1021/acs.nanolett.1c01818)
Supplement: Supplementary file 1 — nl1c01818_si_001.pdf [file nl1c01818_si_001.pdf]

Supporting Information

Optical and magneto-optical properties of donor-bound excitons in vacancy-engineered colloidal nanocrystals

Francesco Carulli[1], Valerio Pinchetti[1], Matteo L. Zaffalon[1], Andrea Camellini[2], Silvia Rotta Loria[2], Fabrizio Moro[1], Marco Fanciulli[1], Margerita Zavelani-Rossi[2], Francesco Meinardi[1], Scott A. Crooker[3] and Sergio Brovelli[1]*

[1]Dipartimento di Scienza dei Materiali, Università degli Studi di Milano-Bicocca, via Cozzi 55, IT-20125 Milano, Italy.
[2]Dipartimento di Energia, Politecnico di Milano and IFN-CNR, Milano, Italy
[3]National High Magnetic Field Laboratory, Los Alamos National Laboratory, Los Alamos, New Mexico 87545, United States

Methods

Chemicals:1-dodecanethiol, DDT, (≥98%), ultrapure water (Chromasolv Plus for HPLC), sodium myristate (≥99%), selenium powder-100 mesh (99.99%), oleic acid, OA (≥90%), cadmium nitrate tetrahydrate (≥98%), 2-propanol (≥99.8%), ethanol (≥99.8%), methanol (≥99.8%), toluene (≥99.5%), 1-octadecene, ODE (≥90%), were purchased from Sigma-Aldrich and gold(III) acetate (≥99.99%) from Alfa Aesar.

Cd-myristate precursor preparation: Cd-myristate precursor was prepared via ex-situ method adding a solution of cadmium nitrate in methanol (0.05 M, 40 mL) a solution of sodium myristate in methanol (0.025 M, 240 mL), rapidly forming a white flocculent precipitate. The white precipitate was washed twice with methanol and dried under vacuum for 24 h to remove all solvents.

Synthesis of V_S:CdSeS NCs: In a 25 mL three-neck flask were subsequently added: 56 mg of Cd-myristate (0.1 mmol), 3.95 mg of Se powder (0.05 mmol), 6.5 mL of ODE, 1 mL of OA and a variable amount of DDT (from 0 to 250 µL). The mixture was degassed under vigorous stirring at room temperature and heated at 80°C with a heating mantle for 1 h. After refilling with N_2, the temperature was raised until nucleation started (i.e. to 160°C for a S:Se=20:1). NCs were grown for 20 minutes, collecting aliquots during particle growth. Aliquots were isolated from the reaction mixture by adding hexane/ethanol mixture (1:3 volume ratio) and then washed twice with hexane/ethanol mixture (1:1 volume ratio). In every step, the NCs were isolated from the supernatant by centrifugation (5000 rpm, 10 min) and re-disperse in hexane for optical studies.

Synthesis of CdSe NCs: In a 25 mL three-neck flask were subsequently added: 56 mg of Cd-myristate (0.1 mmol), 3.95 mg of Se powder (0.05 mmol), 6.5 mL of ODE and1 mL of OA. The mixture was degassed under vigorous stirring at room temperature and heated at 80°C for 1 h. After refilling with N_2, the temperature was raised to 210°C. CdSe NCs were separated and purified with the same procedure adopted for V_S:CdSeS NCs.

Synthesis of CdS NCs: The synthesis of CdS NCs was performed via hot injection method. Specifically, 51.2 mg of CdO (0.4 mmol) were added into a 25 ml three-neck flask with 13.2 mL of ODE and 1 mL of OA. Separately, in an air-free glovebox, 6.4 mg of sulfur powder were dissolved in 2 mL of degassed ODE and heated under stirring at 160°C until transparency, indicating the formation of S-ODE. The CdO mixture was degassed at 100°C for 1 h, refilled with N_2 and subsequently heated to 280°C until the complete dissolution of CdO (color changing from brownish to transparent). The S-ODE solution was quickly injected in the flask and the temperature was rapidly lowered to 250°C and maintained for 15 minutes to complete the NCs growth. CdS NCs were separated and purified with the same procedure adopted for V_S:CdSeS NCs.

Resurfacing through c-ALD technique: c-ALD technique was used to perform a sulfur resurfacing of V_S:CdSeS NCs. In this procedure, the addition of a cadmium sulphide layer on the NCs surface is performed via a two-step reaction. The first step consists in the deposition of a S layer on the NCs and for this purpose 1 mL of toluene, 60 µL of V_S:CdSeS NCs toluene solution (20 mg/ml) and 60 µL of DDABr toluene solution (0.1 M) (non-polar phase), were mixed under vigorous stirring with 1 mL of FA and 30 µL of $(NH_4)_2S$ solution in FA (0.1 M) (polar phase). After 10 mins of stirring the polar phase was removed and substituted with fresh FA to wash the non-polar phase containing DDA^+ stabilized NCs-S^{2-}. In the second step 30 µL of $Cd(OA)_2$ toluene solution (0.1 M) was added to the vial and kept under stirring for 5 mins. The CdS resurfaced NCs were then purified via precipitation with ethanol and re-disperse in toluene for spectroscopical study.

High-Resolution Transmission Electron Microscopy: HR-TEM images were collected with a JEOL JEM-2200FS microscope equipped with a field emission gun working at an accelerating voltage of 200 kV, a CEOS spherical aberration corrector of the objective lens, allowing to reach a spatial resolution of 0.9 Å, and an in-column Omega filter.

Powder X-ray Diffraction: Powder XRD patterns were acquired in Bragg−Brentano geometry with Cu Kα radiation (Panalytical X-Pert Pro powder diffractometer).

Optical characterization: Optical absorption spectra were recorded at room temperature on a Varian Cary 50 Scan UV−visible spectrophotometer under normal incidence in glass cuvettes (optical path 1mm). PL measurements were performed using a pulsed diode laser at 3.05 eV (Edinburgh Inst. EPL 405, 40 ps pulse width) as excitation source and collecting the emitted light with a TM-C10083CA Hamamatsu Mini-Spectrometer. Time-resolved PL measurements were performed using the same excitation source and

collecting with a Hamamatsu R943-02 time-correlated single-photon counting unit coupled to an Oriel Instruments Cornerstone 260 monochromator. All PL measurements were performed with a power density of 100 nJ cm^{-2}. For the ultrafast transient transmission measurements, the laser source was a Ti:sapphire laser with chirped pulse amplification (Coherent LIBRAHE), which provided 95 fs pulses at 1.55 eV at a repetition rate of 2 kHz. The 3.1 eV excitation source was generated by second harmonic generation of the fundamental beam. The bandwidth of the pump pulses is 10 nm which correspond to a pulse duration ~100 fs. The probe beam was a white light supercontinuum generated by focusing a small fraction of the fundamental beam onto a 2mm thick sapphire plate. Pump and probe beams were focused on the sample by means of a lens and a spherical mirror. The excitation fluence used for the transient transmission measurements was ~3 μJ cm^{-2} corresponding to an average exciton population <0.01. A computer-controlled optical multichannel analyzer acquired the map of the differential transmission $\Delta T/T = (T_{on} - T_{off})/T_{off}$, as a function of the pump–probe time delay; T_{on} and T_{off} are the probe spectra transmitted in excited and unperturbed condition respectively.

Spectro-electrochemical measurements: ITO-coated glass slides (R_S < 100 Ω) were purchased from Delta Technologies. Initially, the ITO-coated substrates were covered with ZnO NPs (Nanograde, ~50 nm diameter) to avoid quenching of NCs emission by fast charge/energy transfer to the ITO. The ZnO NP layer (~60 nm thick, measured using a Dektak profilometer) was deposited dip-coating the ITO-coated substrates into an ethanol suspension of ZnO NPs (2 mg ml^{-1}) and annealed at 150°C for 10 min in a nitrogen glovebox. We performed control experiments on the glass/ITO/ZnO NP during potential scans to test the substrates stability, by monitoring the changes of absorption spectra for prolonged exposures to negative and positive potentials. The results of these measurements indicate that the substrates are unaffected by either positive or negative EC potentials for exposure times of tens of minutes (longer than the measurement time in our SEC experiments). The ZnO NP layer used in this study was not treated with crosslinkers and therefore it represented a dielectric tunneling barrier of ~1 V. The NCs were deposited onto the substrate by dip-coating from a dilute toluene solution to form a few-monolayer-thick film. The ITO was connected as a working electrode to a potentiostat (Bio Logic SP-200 Research grade potentiostat/galvanostat) and the film was placed into a quartz cuvette filled with the electrolyte (0.1 M tetrabutylammonium perchlorate ($TBAClO_4$) in propylene carbonate). Ag and Pt wires were used as quasi-reference and counter electrodes, respectively. All potentials reported in this work were measured relative to the quasi-reference Ag electrode during staircase voltammetry scans (10 s scan rate). The film was excited at 3.05 eV with continuous-wave diode lasers and the emitted light was collected with a focusing lens and sent to a spectrometer coupled to a TM-C10083CA Hamamatsu Mini-Spectrometer.

Electron paramagnetic resonance measurements: The EPR spectra were recorded at room temperature on a concentrated solution of NCs with a Varian E15 spectrometer and a Bruker super high Q cavity operated at X-band, ~9.37 GHz, in the absorption mode. The static magnetic field was modulated at 100 kHz with an amplitude of 8 Gauss. The microwave frequency ν_0 and the magnetic field B_0 were monitored continuously with an electronic counter and a Hall probe, and the microwave power was set at 60 mW. The microwave power has been checked not to cause saturation on the observed EPR signal. DPPH was used as reference to determine the *g*-factor.

Temperature- and circular polarization-resolved PL measurements: Low temperature magneto-PL measurements were performed on drop cast films on glass substrates, inserted in the variable temperature insert (5–300 K) of a closed-cycle ^{4}He-cooled split-coil 5 T magnet with direct optical access. The PL was excited at 3.05 eV using an EPL 405 40-picosecond pulsed diode laser by Edinburgh Instruments, and collected with an optical fiber coupled to a TM-C10083CA Hamamatsu Mini-Spectrometer. Time-resolved PL measurements were performed using the same excitation source triggering a Hamamatsu R943-02 time-correlated single-photon counting unit coupled to an Oriel Instruments Cornerstone 260 monochromator. For the circular polarization PL experiments, the helicity of the emitted light was selected by varying the angle between the optical axes of a quarter wave plate and a Glan–Thompson linear polarizer.

Magnetic Circular Dichroism measurements: MCD measures the (normalized) difference in transmission between right- and left-circularly polarized light through the NC film in the Faraday geometry, $(T_R-T_L)/(T_R+T_L)$. The NC films were mounted in the variable-temperature insert (1.5–300 K) of a 7 T superconducting magnet with direct optical access. Probe light of tunable wavelength was derived from a Xenon lamp directed through a spectrometer. The probe light was mechanically chopped at 137 Hz and was modulated between right- and left-circular polarizations at 50 kHz using a photo-elastic modulator. The transmitted light was detected with a silicon avalanche photodiode.

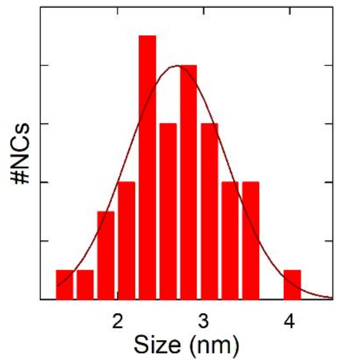

Supporting Figure S1: Size distribution of a representative sample of V_S:CdSeS NCs. The average size is 2.9±0.7 nm.

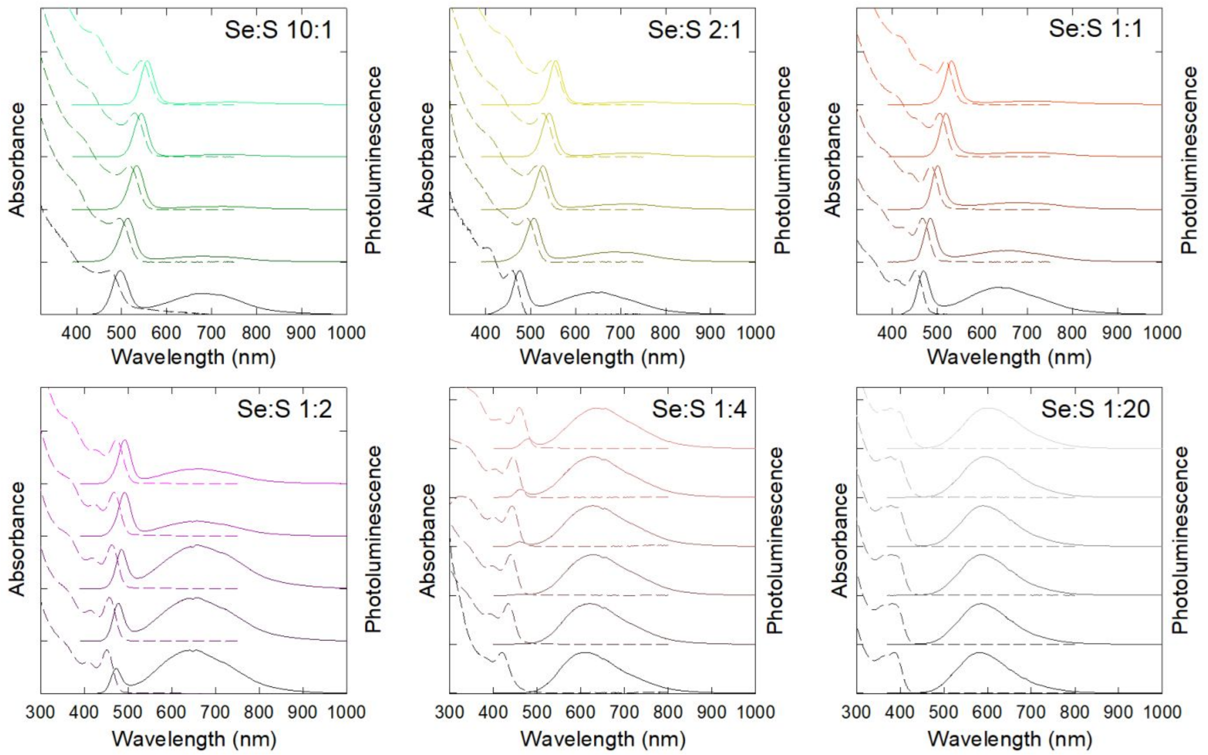

Supporting Figure S2: Optical absorption and PL spectra (dashed and continuous lines, respectively) of V_S:CdSeS NCs prepared with different Se/S ratio (from 10:1 to 1:20) collected at different synthesis times: 30s, 1 min, 3 min, 5 min, 7 min and 10 min from bottom to top, respectively.

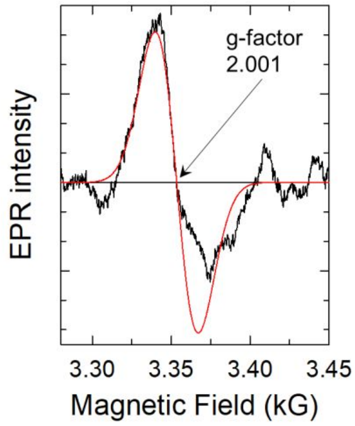

Supporting Figure S3: Electron paramagnetic resonance of V_S:CdSeS NCs recorded at 9.37 GHz frequency (black curve) and respective fitting curve (red curve) with a single Gaussian resonance peak featuring g=2.001±0.001.

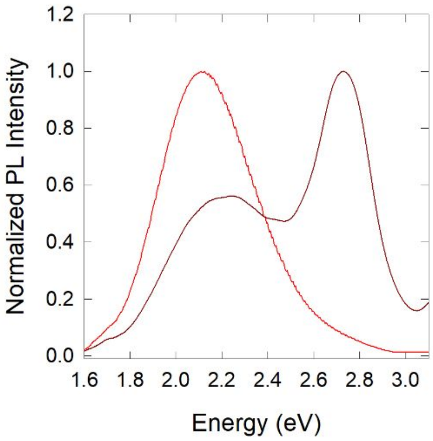

Supporting Figure S4: Photoluminescence spectra of V_S:CdSeS NCs before (red curve) and after (black curve) c-ALD treatment, showing the emergence of the band edge luminescence at ~2.75 eV upon surface sulfuration.

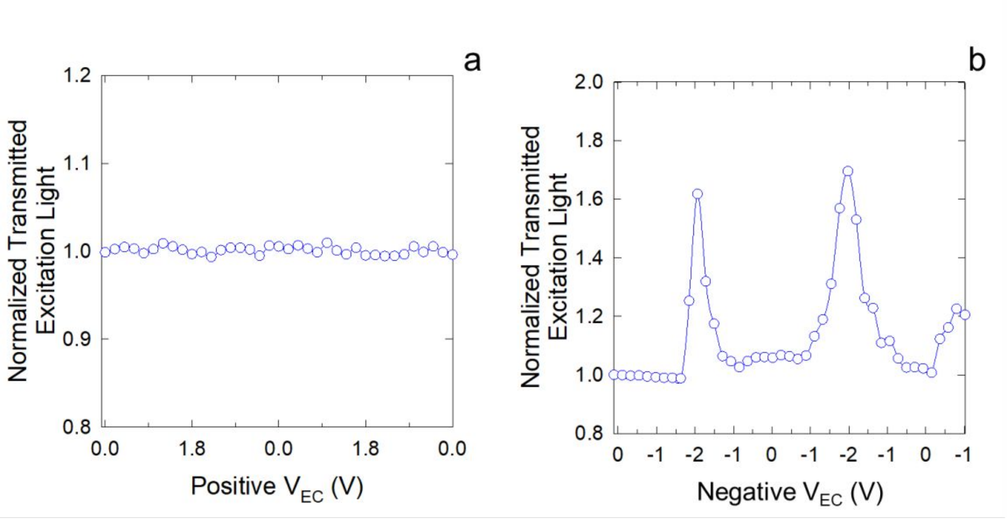

Supporting Figure S5: Normalized intensity of the transmitted excitation light at 3.05 eV used in SEC measurements as a function of the applied voltage (V_{EC}) for two (a) positive and (b) negative scans.

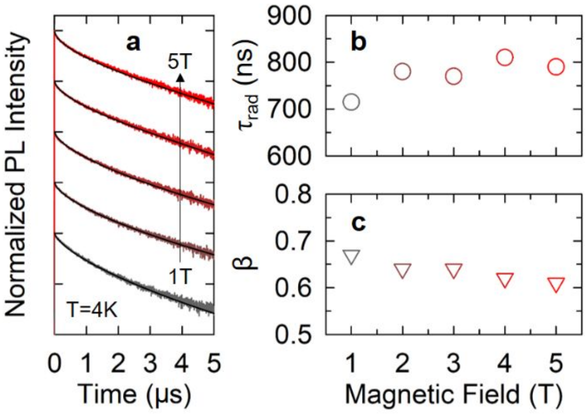

Supporting Figure S6: (a) Normalized time-resolved PL decay curves of V_S:CdSeS NCs at T=4 K and increasingly more intense B from B=1 T (grey curve) to B=5 T (red curve). Black lines are the respective fitting curves with the equation $I(t)=I_0 \cdot exp(-(t/\tau_{rad})^{\beta})$, where I_0 is the zero-delay PL intensity, τ_{rad} is the characteristic PL decay lifetime and β is the stretching factor. (b) τ_{rad} and (c) β dependence on the magnetic field showing nearly constant values in both cases.

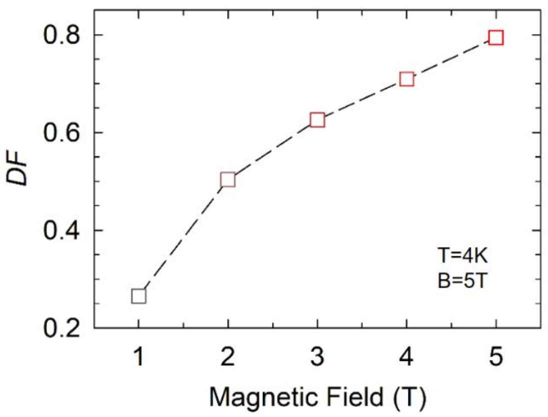

Supporting Figure S7: Magnetic field dependence of the dynamical factor (*DF*) defined as $\tau_{rad}/(\tau_{rad}+\tau_{spin})$ and calculated using the experimental data reported in **Fig.4f**.

Supporting Discussion

Evaluation of the average number of vacancies per NCs.

In order to evaluate the average number of vacancies per NC, we compared the intensity of the V_S and BE emission contribution for NCs batches prepared with different S/Se ratio. Assuming that every NC with at least one V_S emits only from the V_S state and within the approximation that doped and undoped particles are equally excited by above-gap excitation, we estimate the fraction of doped particles (f_{V_S}), through the expression:

$$f_{V_S} = \frac{\left[\Phi_{V_S-PL}\int PL_{V_S}(E)dE\right]}{\left[\Phi_{V_S-PL}\int PL_{V_S}(E)dE + \Phi_{BE-PL}\int PL_{BE}(E)dE\right]},$$

where the integrated intensity of the BE and V_S emission bands are weighted for the respective room temperature PL quantum yield measured for unalloyed ($\Phi_{BE\text{-}PL}$=35±4%) and fully self-doped NC samples ($\Phi_{V_S\text{-}PL}$=17±2%). The fraction of NCs without any V_S (i.e. 1-f_{V_S}) was then used to estimate the average number of vacancies in the approximation of a Poissonian distribution of vacancies in the NCs ensemble. With this model, estimate that in a NC ensemble having f_{V_S}>0.99 (i.e. fully vacancy-doped ensembles), each NC contains an average of seven sulfur vacancies

Theoretical calculation of P_C^{eq} and P_C^{int} for an ensemble of randomly oriented spherical NCs.

Assuming a Boltzmann population distribution of the VB hole spin sublevels split by an external magnetic field B, the intensity of the circularly-polarized PL bands can be written as[1]

$$I^{\pm}=1+x^2\mp 2x\rho_0(B,x,T) \qquad \text{(S1)}$$

where x=cosθ, with θ being the angle between the direction of the external magnetic field and the quantization axis of the individual nanocrystal and[2]

$$\rho_0(B,x,T)=\tanh[\Delta E_Z(B,x)/2k_BT] \qquad \text{(S2)}$$

with ΔE_Z=-3$g\mu_B$Bx, since VB holes are featured by |M|=3/2. Hence, by substituting **Eq.S1** into **Eq.5**, we can express the equilibrium polarization degree P_C^{eq} as

$$P_C^{eq}(B,x,T)=-2x\rho_0(B,x,T)\,/(1+x^2) \qquad \text{(S3)}$$

and, considering a random orientation of the nanocrystals due to their almost perfect spherical shape, we can integrate **Eq.S3** over x, obtaining

$$P_C^{eq}(B,T)=-(\int_0^1 2x\rho_0(B,x,T)dx)/(\int_0^1 1+x^2dx) \qquad \text{(S4)}$$

We highlight that, in thermal equilibrium conditions, the observed circular polarization P_C^{eq} is only determined by ΔE_Z and temperature. Contrarily, the time-integrated circular polarization P_C^{int} also depends on the spin-flip time, since the early-times PL decay accounts for the most-intense the PL signal with the lowest-intense circular polarization degree. This disproportion is well quantified by the dynamical factor, *DF*, introduced in the main text. P_C^{int} can be therefore written as

$$P_C^{int}(B,T)=-(\int_0^1 2x\rho_0(B,x,T)\mathit{DF}dx)/(\int_0^1 1+x^2dx) \qquad \text{(S5)}$$

Finally, it is worth noting that, explicating **Eq.S2** and ΔE_Z and considering B and T as known experimental parameters, **Eq.S4** and **Eq.S5** are only featured by one single fitting parameter, i.e. the *g*-factor responsible for the Zeeman splitting.

Supporting References

1. Efros, A. L.; Rosen, M.; Kuno, M.; Nirmal, M.; Norris, D. J.; Bawendi, M. *Physical Review B* **1996,** 54, (7), 4843-4856.
2. Liu, F.; Biadala, L.; Rodina, A. V.; Yakovlev, D. R.; Dunker, D.; Javaux, C.; Hermier, J.-P.; Efros, A. L.; Dubertret, B.; Bayer, M. *Physical Review B* **2013,** 88, (3), 035302.
